# Disseminated Granulomatous Disease from Intravesical Instillation of Bacillus Calmette-Guerin

**DOI:** 10.1155/2018/8280527

**Published:** 2018-08-29

**Authors:** Sayed Ab. Reshad Ghafouri, Alexander Brun, Rohit Bhalla, Craig Margulies, Kevin Skole

**Affiliations:** ^1^Triborough GI Gastroenterology, NY Scientific Research Center, 1517 Voorhies Avenue, Brooklyn, NY 11235, USA; ^2^Mount Sinai Beth Israel, 3201 Kings Highway, Brooklyn, NY 11234, USA; ^3^ID Care, 105 Raider Blvd. Suite 101, Hillsborough, NJ 08844, USA; ^4^Digestive Disease Center, 11 State Road, Suite 200, Princeton, NJ 08540, USA; ^5^University of Medicine and Dentistry of New Jersey, Robert Wood Johnson Medical School, University Medical Center at Princeton, 253 Witherspoon Street, Lambert House, Princeton, NJ 08540, USA

## Abstract

Intravesical instillation of Bacillus Calmette-Guerin is one of the standard treatment options for superficial bladder cancer. While Bacillus Calmette-Guerin therapy is usually well tolerated with most patients experiencing only cystitis, in rare cases, it can lead to disseminated granulomatous disease. We present a case of a 72-year-old man with disseminated granulomatous disease from intravesical BCG instillation whose treatment was complicated by antimycobacterial drug toxicity.

## 1. Introduction

Bacillus Calmette-Guerin (BCG) is a viable strain of the virulent *Mycobacterium bovis*. It was developed at the end of the 19^th^ century and has been used extensively as a vaccine against tuberculosis since the 1920s [1]. In 1976, Morales et al. first reported that instillation of BCG could be used as an alternative to chemotherapy in the treatment of in situ transitional cell carcinoma of the bladder [2]. Since then, BCG has become a commonly used treatment with proven efficacy.

The induction of local inflammation makes hematuria, cystitis, and transient fevers a common and almost an inherent part of the BCG treatment [3, 4]. Systemic complications from BCG therapy, such as pneumonitis, granulomatous hepatitis, and sepsis, are rare and usually occur after traumatic and difficult bladder catheterizations [5, 6]. We present a case of disseminated granulomatous disease secondary to BCG treatment, complicated by antimycobacterial drug toxicity.

## 2. Case

A 72-year-old male was admitted with anorexia, shaking chills, diaphoresis, hematuria, productive cough, and fever up to 103°F.

Ten months prior to his presentation, the patient had been diagnosed with high-grade papillary noninvasive urothelial carcinoma of the bladder with no invasion of the lamina propria or muscularis propria (American Joint Committee on Cancer stage TaN0M0). After the tumor was resected, he began intravesical BCG therapy. He had his final BCG instillation approximately 17 days prior to his admission. On the day of admission, he had significant gross hematuria, shaking chills, a productive cough, profuse diaphoresis, malaise, and fever up to 103°F.

The patient's past medical history included type 2 diabetes mellitus, hypertension, and coronary artery disease. Medications on admission were losartan, INH insulin, metformin, metoprolol, and rosuvastatin. The patient was originally from India and had lived in the United States for the past thirty years. He had a 15-pack year smoking history prior to quitting 25 years ago. His family history was significant for prostate cancer in his brother.

Upon admission, the patient appeared fatigued and acutely ill. His temperature was 103°F, respiratory rate 24, blood pressure 160/80, pulse 84, and pulse oximetry 97% on 2 L nasal canula. His exam was notable for bilateral crackles at the lung bases; his abdomen was benign, and there was no costovertebral angle tenderness.

Laboratory results included a white blood cell count of 3.6 × 10^9^/L, hemoglobin of 14.4 g/dL and platelet count of 98 × 10^9^/L. Liver function tests showed an alkaline phosphatase of 251 U/L, alanine transaminase of 71 U/L, and aspartate transaminase of 92 U/L. Urinalysis had moderate blood, 3–10 RBC/hpf, 0–5 WBC/hpf, negative leukocyte esterase, and negative nitrites. PA and lateral chest X-ray showed increased interstitial markings bilaterally, especially at the bases. Right upper quadrant ultrasound showed mild fatty infiltration of the liver.

The patient was placed on ceftriaxone for presumed community-acquired pneumonia but failed to improve. Lack of improvement on antibiotics coupled with the multiple systemic abnormalities (including (1) chest X-ray findings suggesting pneumonitis, (2) a rise in transaminases pointing towards hepatitis, and (3) thrombocytopenia indicating bone marrow involvement) lead to the possible diagnoses of disseminated mycobacteria. However, it was not clear whether this was truly disseminated mycobacteria or a hypersensitivity reaction from BCG. Therefore, the patient was initially started on methylPREDNISolone, INH, rifampin, and ethambutol.

The patient began to improve clinically, but after six days of therapy, his transaminases were markedly more elevated with worsening thrombocytopenia. At this point, concurrent medication toxicity was suspected. His antimycobacterial medications were held, and both a liver biopsy and a bone marrow biopsy were obtained in the following two days. His transaminases returned began to decrease shortly after antimycobacterial medications were held. Rifampin and ethambutol were restarted 24 hours later.

**Figure 1:**
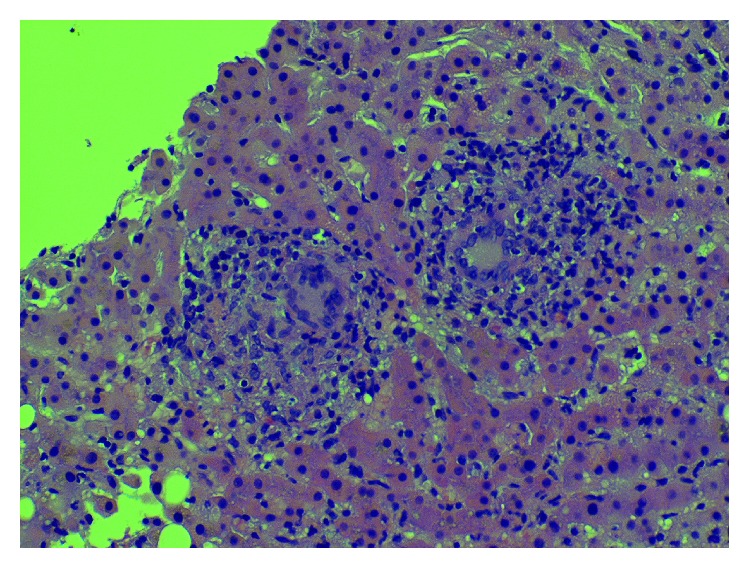
Pathology slide of the liver showing noncaseating granulomas.

The liver biopsy showed chronic lobular and portal inflammation consisting predominantly of lymphocytes and histiocytes, focal interface hepatitis, and small noncaseating granulomas (see Figure 1). The bone marrow biopsy showed scattered noncaseating granulomas. Acid-fast stain for mycobacteria was negative on both biopsies. All blood cultures, urine cultures, and sputum cultures were ultimately negative.

The patient was discharged on rifampin, ethambutol, and prednisone eight days after the biopsy results came back. In the following week, he developed a recurrent rise in his transaminases, leading to cessation of antimycobacterial therapy due to possible rifampin hepatotoxicity. He remained stable with normal liver enzymes after completing six weeks of steroid treatment.

Based on the aforementioned findings, the diagnosis of systemic granulomatous disease from BCG therapy was established, with hypersensitivity being the most likely etiology.

## 3. Discussion

While treatment of superficially invasive transitional cell carcinoma of the urinary bladder with BCG is considered effective and safe, local side effects are common (up to 90%), and systemic complaints can occur in up to 3% of patients [6, 7]. Risk factors for serious complications include traumatic catheter insertion, repeat instillation of BCG in patients with persistent local side effects (e.g., cystitis), and comorbidities [3, 8].

It is recommended not to give BCG therapy within the first 14 days of bladder surgery, and the BCG should not be given after a traumatic catheterization as it provokes disseminating BCG infection [9]. Systemic administration of antimycobacterial drugs was shown to be effective as the BCG strains are sensitive for most tuberculostatic medications. Two doses of 200 mg ofloxacin given shortly after BCG instillation have been demonstrated to reduce moderate to severe side effects [10]. Low dose BCG was tried in several studies in an attempt to decrease the frequency and severity of side effects and were found to be significantly less compared to full dose BCG treatment [11].

Our patient, a diabetic, was having more widespread inflammatory response after his last BCG instillation. At issue is whether this response, i.e., systemic granulomatous disease, is a result of sepsis, a hypersensitivity reaction, or both.

All cultures in our patient were negative. It is notable, however, that with early disseminated granulomatous disease (defined as occurring within 8–12 weeks after the first BCG treatment) direct cultures, acid-fast smears and even DNA hybridization of granulomatous tissue infrequently demonstrate clear mycobacterium; blood cultures and bone marrow evaluations are also rarely positive [12]. The low yield is presumed to be due to an immunocompetent host forming well-developed granulomas without detectable organisms [12, 13]. In a study that combined results of 20 patients with early presentation, culture results were positive in 30% [12]. Lack of identifiable organisms in the majority of cases supports the theory that early disseminated granulomatous disease is more often a hypersensitivity reaction, and therefore, treatment with corticosteroids is advocated [13, 14]. In contrast, among patients with late presentation, cultures yield a positive result in 67%. The likely mechanism may be due to the reactivation of controlled early dissemination of *Mycobacterium bovis* now leading to active infection [12]. There have been no reported trials to assess optimal therapy for disseminated granulomatous disease. The absence of data, coupled with the fact that *Mycobacterium bovis* has been isolated from the tissue in both early [15] and late presentations [16], leads to the conclusion that corticosteroids should be combined with an antimycobacterial drug regimen until biopsy results are complete.

Our patient was started on both an antimycobacterial regimen and systemic anti-inflammatory treatment. Soon after treatment was started, however, his transaminases increased sharply. They returned to their previous levels when antimycobacterial therapy was stopped.

Mild elevation of transaminases (<3x upper limit of normal) occurs in up to 20% of patients taking INH [17, 18]. Less than 1% develop severe hepatitis [17, 19]. The etiology of INH hepatotoxicity is unclear. Several studies suggest it is a direct result of toxic metabolites, while others support the theory that toxicity results from slow acetylation of the drug leading to the use of alternative, more toxin-inducing pathways [20, 21]. INH toxicity is difficult to diagnose by biopsy since the pathologic process ranges from mild patchy hepatitis to bridging and multilobular necrosis to severe hepatic necrosis [22, 23]. The biopsy results in our patient, including a lymphocyte predominance, seem to support the combination of acute drug toxicity and granulomatous hepatitis as the cause of the acute increase in our patient's liver enzymes. An eosinophil predominance would be expected in a pure hypersensitivity reaction [24].

INH-induced hepatitis generally occurs 4 to 8 weeks after the start of therapy, but a more rapid onset has been well-documented [22, 25]. Age appears to be the most important risk factor of INH-induced hepatitis. Hepatic damage is rare in those less than 20 years of age, occurs in 1.2% of patients who are 35 to 49, and the incidence rises even further to 2.3% in people over 50 [26, 27]. Our patient was well over 50 years old. In addition, he was initially treated with a combination of INH and rifampin, which has been shown to lead to more rapid development of hepatitis in comparison to INH alone [28]. Rifampin increases hydrazine by induction of isoniazid hydrolase, which may explain the severe hepatotoxicity [29].

The recurrence of elevated transaminases one week post discharge is attributed to rifampin hepatotoxicity. Studies looking at patients taking prophylactic rifampin monotherapy report hepatotoxicity in about 1–2% [30, 31]. The pathophysiology of rifampin-induced hepatotoxicity is unknown and usually unpredictable. Hepatitis from hypersensitivity to rifampin has been reported but only in isolated case reports [32].

Guidelines of the American Thoracic Society for management of antimycobacterial drug hepatotoxicity recommend continuation of medication when hepatotoxicity is mild [33]. However, all drugs should be discontinued if ALT rises to five times the normal, since continuation after signs of severe hepatic dysfunction develop tends to increase the severity of liver damage [33, 34]. Once ALT normalizes (ALT < 2 times upper limit of normal), rifampin (with or without ethambutol) should be restarted. If symptoms recur or ALT increases, rifampin is to be ceased.

Our patient presented with elevated transaminases and ultimately had biopsy-proven disseminated granulomatous disease. He significantly improved with steroids after cessation of antimycobacterium therapy which is consistent with a hypersensitivity reaction to intravesical BCG therapy.

## 4. Conclusion

We present a case of disseminated granulomatous disease secondary to intravesical BCG therapy for superficial bladder cancer. His treatment was complicated by presumed INH and rifampin hepatotoxicity. BCG therapy is considered an excellent alternative to chemotherapy in the treatment of superficial bladder tumors. Side effects are common, however, and systemic complications can occur. The exact mechanism of disseminated granulomatous disease is unclear, and treatment includes both antimicrobial and anti-inflammatory therapy.
